# 离子色谱法同时检测大气颗粒物PM_2.5_中低级脂肪胺和常规阳离子

**DOI:** 10.3724/SP.J.1123.2023.10029

**Published:** 2024-05-08

**Authors:** Dandan ZHANG, Shuang ZHU, Chang HOU, Danni CAI, Guangli XIU, Shaorong LUAN

**Affiliations:** 1.华东理工大学化学与分子工程学院, 上海 200237; 1. School of Chemistry and Molecular Engineering, East China University of Science and Technology, Shanghai 200237, China; 2.华东理工大学资源与环境工程学院, 国家环境保护化工过程环境风险评价与控制重点实验室, 上海 200237; 2. State Environmental Protection Key Laboratory of Environmental Risk Assessment and Control of Chemical Process, School of Resources and Environmental Engineering, East China University of Science and Technology, Shanghai 200237, China

**Keywords:** 离子色谱, 低级脂肪胺, 阳离子, PM_2.5_, ion chromatography (IC), lower aliphatic amines, cations, PM_2.5_

## Abstract

大气中存在大量的有机胺污染,其中低级脂肪胺是促进颗粒形成和生长成为PM_2.5_的诱因,会对人体肾脏、心肺功能健康造成损害。而PM_2.5_是大气中常见的颗粒污染物,是雾霾天气产生的主要原因,其成分非常复杂,测定其中的阳离子和低级脂肪胺,能直接监测环境大气质量,保护人体健康。本研究建立了抑制电导离子色谱法同时测定大气细颗粒物PM_2.5_中4种低级脂肪胺(甲胺、二甲胺、三甲胺、乙胺)和5种常见阳离子(Na^+^、N $H_{4}^{+}$ 、K^+^、Mg^2+^、Ca^2+^),通过优化色谱条件,实现了K^+^和甲胺、二甲胺和乙胺等难分离物质的有效分离,分析结果可用于评估空气中的颗粒物污染情况。本研究采用负载石英滤膜的中流量采样器采集大气中的PM_2.5_颗粒物,裁剪1/2滤膜并剪碎于10 mL超纯水中超声提取2次共60 min,提取液过0.22 μm滤膜后用离子色谱检测。比较了3种阳离子色谱柱IonPac^TM^ CS17、IonPac^TM^ CS16和SH-CC-9后,最终选用SH-CC-9阳离子分析柱(200 mm×4.6 mm)进行分离:柱温30 ℃,检测器温度35 ℃,进样量25 μL,流动相为甲基磺酸(MSA)水溶液,流速为1.1 mL/min。在此色谱条件下,大气中可能存在的其他胺(*N*,*N*-二甲基甲酰胺、*N*,*N*-二甲基乙酰胺、丙胺、二乙胺、三乙胺、三乙醇胺、异丙胺)对目标离子没有影响。待测的4种低级脂肪胺和5种常见阳离子在各自相应的浓度范围内呈现良好的线性关系,线性相关系数(*r*)均不小于0.997,检出限(LOD)为0.02~1.90 μg/L,定量限(LOQ)为0.07~6.32 μg/L, 6个平行样品测定的相对标准偏差(RSD)小于2%,样品加标回收率为93.2%~104%。采用建立的方法对189个大气颗粒物PM_2.5_样品进行检测,9种离子均有检出,其中Na^+^、N $H_{4}^{+}$ 、Ca^2+^等离子含量较多,4种低级脂肪胺含量少,但部分样品中乙胺含量偏高。结果表明,本研究建立的离子色谱方法前处理简单,灵敏度高,准确性好,可满足大气颗粒物PM_2.5_中低级脂肪胺和常规阳离子同时定量检测的需求,能够快速处理大量样品,准确评价空气中颗粒物的污染程度,追溯污染来源,保护人类健康。

## Abstract (en)

Many amine pollutants exist in the atmosphere. Lower aliphatic amines promote the formation and growth of particles into PM_2.5_, which damages the heart, lungs, and kidneys of the human body. PM_2.5_, a common atmospheric particulate pollutant with complex compositions, is the main cause of haze weather. Therefore, measuring the contents of lower aliphatic amines and cations in PM_2.5_ is of great significance for monitoring environmental air quality and protecting human health. This study established a suppressed ion-chromatographic method with conductivity for the simultaneous detection of four lower aliphatic amines (methylamine, dimethylamine, trimethylamine, and ethylamine) and five cations (Na^+^, N $H_{4}^{+}$ , K^+^, Mg^2+^, and Ca^2+^) in PM_2.5_. The effective separations of several groups of substances that are difficult to separate, such as K^+^ and methylamine, dimethylamine, and ethylamine, were achieved by optimizing the chromatographic conditions. The results were then used to assess the pollution caused by small particles in the air. A medium-flow sampler loaded with a quartz filter membrane was used to collect PM_2.5_ particles in the atmosphere. The sample flow rate was 100 L/min, and the sampling time was 24 h. Half of the filter membrane was cut and shredded into 10 mL of ultrapure water for two cycles of ultrasonic extraction over 60 min. After membrane filtration, the extract was analyzed using ion chromatography (IC). Three cation-exchange columns (IonPac^TM^ CS17, IonPac^TM^ CS16, and SH-CC-9) were compared, and the SH-CC-9 column (200 mm×4.6 mm) was ultimately used for separation under a column temperature of 30 ℃, detector temperature of 35 ℃, and injection volume of 25 μL. An aqueous solution of methyl sulfonic acid (MSA) was used as the mobile phase at a flow rate of 1.1 mL/min. Under these conditions, other aliphatic amine ions in air (triethanolamine, diethylamine, triethylamine, isopropylamine, propylamine, *N*,*N*-dimethylformamide, and *N*,*N*-dimethylacetamide) did not interfere with the separation and detection of the nine target ions. The nine ions showed good linear relationships within the corresponding concentration range, with linear correlation coefficients (*r*) no less than 0.997. The limits of detection (LODs) were 0.02-1.90 μg/L, and the limits of quantification (LOQs) were 0.07-6.32 μg/L. The relative standard deviations (RSDs) for the determination of six parallel samples were less than 2%, and the sample recoveries were between 93.2% and 104%. The established IC method was used to detect 189 PM_2.5_ samples collected from the atmosphere, and all nine ions were successfully detected. Among these ions, Na^+^, N $H_{4}^{+}$ , and Ca^2+^ showed high concentrations. The contents of the four lower aliphatic amines were low; however, the ethylamine content in some samples was high. The results indicate that the proposed method meets the quantification requirements for cations and lower aliphatic amines in PM_2.5_, with simple processing, high sensitivity, and good accuracy. It can quickly and accurately detect a large number of samples and be used to assess the pollution of small particles in the air as well as trace pollution sources to protect human health.

雾霾是全球性的空气污染问题,颗粒污染物是其主要组成。颗粒污染物依据空气动力学直径的大小可以分为PM_10_、PM_2.5_、PM_1_。其中PM_2.5_主要是指粒径小于2.5 μm的颗粒物,主要成分包括有机碳和无机碳组分、水溶性离子组分以及其他化合物。PM_2.5_直径小,可进入人体支气管,成为有毒有害物质的沉积载体和催化剂^[1]^,损害肾脏,诱发肾功能下降^[2,3]^,导致呼吸系统疾病^[4,5]^、心血管疾病^[6,7]^、神经系统疾病^[8]^,甚至癌症^[9,10]^等。城市PM_2.5_污染的主要来源是汽车尾气排放、工业废气^[11]^等。《环境空气质量标准》^[12]^对PM_2.5_的年平均限值规定为15 μg/m^3^。

脂肪胺是氨的烷基取代衍生物,是大气中重要的碱性成分,其中短链烷基胺是主要的胺形式。胺是某些致癌致病物质的前体,例如大气中的脂肪胺可以通过光氧化还原反应生成亚硝胺和硝胺等致癌物^[13,14]^,对人体健康产生危害。同时气态低级脂肪胺可以通过酸碱反应、凝结成液滴进入颗粒相,促进大气新颗粒相的成核、生长,逐渐形成气溶胶。通过改变气溶胶的密度、吸湿性等性质,对气候的变化产生影响^[15,16]^。因此,建立简单、快捷、准确的方法检测大气颗粒物中的低级脂肪胺含量,对评估大气中的颗粒物污染情况意义重大。

大气颗粒物中的有机胺含量低,种类多,给检测带来很大困难。目前主要检测方法有高效液相色谱-质谱法、气相色谱-质谱法、离子色谱法(IC)等。气相色谱-质谱法和高效液相色谱-质谱法都需要将样品进行衍生化处理,操作过程复杂^[17⇓⇓-20]^,导致结果重复性不理想。与前两种方法相比,IC前处理简单,灵敏度高^[21⇓-23]^。彭绪玲等^[24]^采用IC在30 min内实现了Na^+^、K^+^、N $H_{4}^{+}$ 、Mg^2+^、Ca^2+^5种阳离子和丙胺(PA)、二甲胺(DMA)、三甲胺(TMA)3种低级脂肪胺的分析检测;环境保护行业标准将离子色谱法作为环境空气中常规阴阳离子及氨、甲胺(MA)等低相对分子质量脂肪胺的推荐检测方法^[25⇓-27]^。但以上离子色谱方法都无法实现常规K^+^和MA、DMA和乙胺(EA)等相对分子质量相近的胺的分离,因此本文建立了一种新的离子色谱检测方法,实现了MA、DMA、TMA、EA 4种短链脂肪胺及5种常见阳离子的同步分析,对研究大气脂肪胺的组成和来源、评估大气污染状况、监测环境空气质量和保护人类健康具有重要意义。

## 1 实验部分

### 1.1 仪器与试剂

ICS-6000型离子色谱仪(美国Thermo Fisher公司),配备Chromeleon 7.2.1色谱工作站、自动进样器(AS-AP)、双四元泵、甲磺酸(MSA)淋洗液自动发生器、Dionex CDRS 600(4 mm)阳离子抑制器和电导检测器;TH-100中流量采样器(武汉天虹环保产业股份有限公司); Milli-Q超纯水机(美国Millipore公司); KQ-200KDE型高功率数控超声清洗器(昆山市超声仪器有限公司);水相针式过滤器(0.22 μm,上海安谱实验科技股份有限公司)。

甲胺、二甲胺、三甲胺、乙胺(分析纯,美国Sigma公司);钠、铵离子单元素标准溶液(1000 mg/L,国家有色金属及电子材料分析测试中心);水中钾、镁、钙分析标准物质(100 mg/L,上海市计量测试研究院)。

### 1.2 色谱条件

SH-CC-9阳离子色谱分离柱(200 mm×4.6 mm,青岛盛瀚色谱技术有限公司); 采用在线甲基磺酸淋洗液自动发生器进行梯度洗脱:0~22 min, 2 mmol/L MSA; 22.1~55 min, 3.5 mmol/L MSA; 55.1~60 min, 2 mmol/L MSA; CDRS 600阳离子抑制器,抑制电流11 mA;进样量25 μL;流速1.1 mL/min;柱温30 ℃;采用电导检测器,检测池温度35 ℃。

### 1.3 标准储备液的配制

分别称取4种有机胺标准品20 mg于50 mL离心管中,加入超纯水溶解并定容至50 mL,配制成400 mg/L的有机胺混合标准储备液。有机胺混合标准储备液和阳离子标准溶液放置于4 ℃药品阴凉柜中避光保存。使用时用超纯水稀释,配制成所需浓度的系列混合标准溶液。

### 1.4 样品采集及预处理

PM_2.5_使用TH-100中流量采样器和直径90 mm的石英滤膜,根据环境空气颗粒物采集相关标准HJ 799-2016、HJ 800-2016、HJ 93-2013要求^[25,26,28]^采集,采样器流速设置为100 L/min,采样时间为24 h。文献[29]中,在此条件下,采样效率大于99.7%,符合PM_2.5_采集滤膜截留效率要求。本实验按照此程序进行实际采样过程中,没有穿透现象发生。

取采集样品后的滤膜1/2,剪碎,置于50 mL离心管中,加入10 mL超纯水,为避免有机胺的挥发,采用冰水浴超声,操作2次,每次30 min。吸取样品溶液,过0.22 μm的水相滤膜,滤液转移至1.5 mL样品瓶进行离子色谱分析。

### 1.5 大气PM_2.5_中阳离子和有机胺含量计算

根据采样器的采样流速和采样时间、采集滤膜的提取体积、离子色谱检测得到的各离子的浓度,采用公式(1)计算大气PM_2.5_中阳离子和有机胺的含量。


(1) $$\rho_{PM2.5}=\frac{\rho_{\text{提取液 }}\times V_{\text{提取体积 }}}{V_{\text{采集体积 }}}$$

式中:*ρ*_PM2.5_为大气PM_2.5_中离子的质量浓度(ng/m^3^); *ρ*_提取液_为PM_2.5_提取液中离子的质量浓度(ng/L); *V*_提取体积_为提取液的体积(L); *V*_采集体积_为采集PM_2.5_的气体总体积(m^3^)。

## 2 结果与讨论

### 2.1 色谱条件优化

#### 2.1.1 色谱柱的选择

空气颗粒物中5种阳离子含量多,脂肪胺含量少,由于这些阳离子和脂肪胺的物理化学性质比较接近,大量特定阳离子的存在会影响到小分子胺的检测,小分子脂肪胺之间的分离也非常困难,这就给色谱分离提出很高要求。本工作分别考察了IonPac^TM^ CS16、IonPac^TM^ CS17以及SH-CC-9阳离子分离柱对目标离子的分离效果。

CS16是一种带有羧酸功能基团的大容量阳离子交换柱,交换能力弱,可以用于分离碱金属、碱土金属和铵离子等。如图1所示,CS16色谱柱在用于脂肪胺的分离时,4种有机胺仅有3个峰出现,甲胺和乙胺峰完全重合;尝试改变淋洗液浓度也没有实现二者的完全分离,所以该CS16色谱柱不适用于PM_2.5_颗粒物中目标离子的分析。

**图1:**
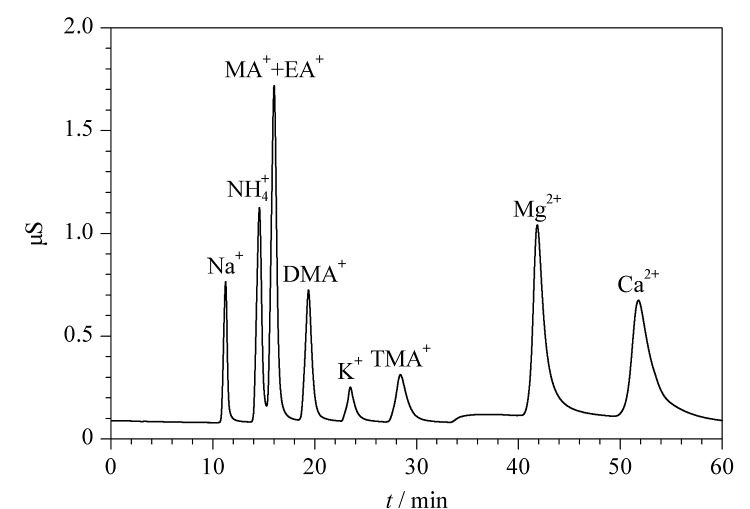
IonPac^TM^ CS16色谱柱分离9种目标阳离子的色谱图

CS17是一种容量中等的阳离子交换柱,可以用于疏水离子或胺、碱金属、碱土金属以及铵离子等的分离,实际分离结果如图2所示。

**图2:**
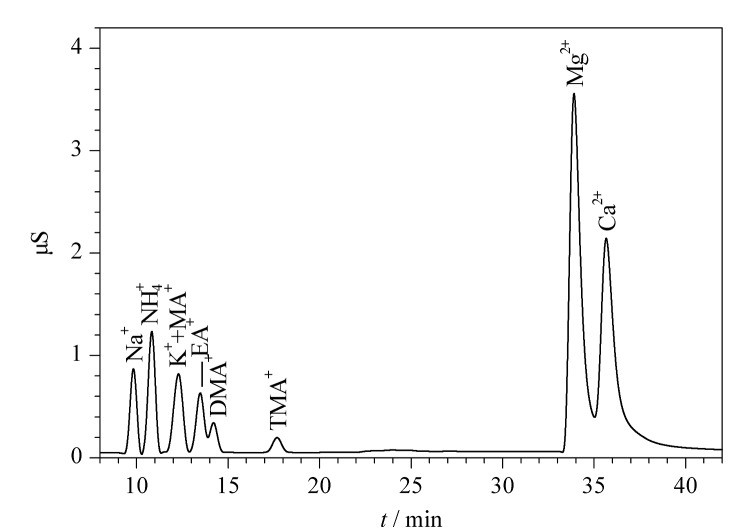
IonPac^TM^ CS17色谱柱分离9种目标阳离子的色谱图

在该色谱条件下,虽然在25 min内3种阳离子和4种脂肪胺能够完全洗脱,但K^+^与甲胺的色谱峰重合为一个峰,且通过调整淋洗液浓度,K^+^与甲胺的色谱峰始终无法有效分离。考虑到PM_2.5_颗粒物中K^+^含量很高,甲胺含量很低,二者浓度相差几百倍以上,所以采用合适的方法将二者完全分离从而得到甲胺含量尤为重要;乙胺与二甲胺的保留性质比较接近,能实现的最好分离度也只有1.08,形成了肩峰,无法实现完全分离,影响到二者的定量结果,所以CS17色谱柱不适用于本研究中PM_2.5_中低级脂肪胺和常规阳离子的分离。

SH-CC-9是一种弱酸型阳离子色谱柱,其基质为交联度55%的苯乙烯-二乙烯苯聚合物,表面接枝羧基,可用非抑制或抑制电导法完成常规阳离子分析,该分离柱可以在较低的淋洗液浓度下一次进样,实现PM_2.5_中钠、铵、钾、镁、钙等常规阳离子以及甲胺、二甲胺、三甲胺和乙胺等脂肪胺的分离,峰形良好,分离度满足要求。如图3所示,乙胺和二甲胺的分离度为2.16, K^+^和三甲胺的分离度为3.05,其余各峰间的分离度均大于3。环境大气颗粒物中可能会存在*N*,*N*-二甲基甲酰胺、*N*,*N*-二甲基乙酰胺、二乙胺(DEA)、三乙胺(TEA)、三乙醇胺(TEOA)、异丙胺(IPA)、丙胺等其他小分子有机胺,与目标离子共洗脱,并对之分离分析产生干扰。

**图3:**
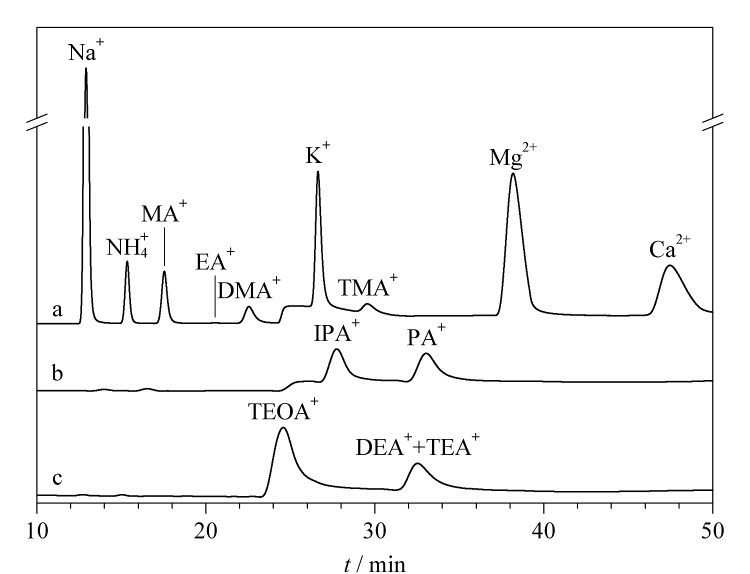
SH-CC-9色谱柱分离9种目标离子及5种可能有机胺的色谱图

在设定的色谱条件下对这些有机胺的洗脱情况进行考察,如图3所示,在SH-CC-9色谱柱上,K^+^、TMA^+^的保留时间分别为26.2、28.9 min,三乙醇胺、异丙胺、丙胺的保留时间分别为24.6、27.7、33.0 min,二乙胺和三乙胺峰保留时间接近(32.6 min), *N*,*N*-二甲基甲酰胺、*N*,*N*-二甲基乙酰胺在设定条件下无法洗脱,所以这7种可能存在的有机胺不会对本研究的9种目标离子产生干扰,因此选择SH-CC-9色谱柱作为分离柱。

#### 2.1.2 梯度洗脱程序的优化

9种离子在SH-CC-9色谱柱上保留性质差异很大,所以选择MSA淋洗液梯度洗脱。Na^+^、N $H_{4}^{+}$ 的保留相对较弱,且保留性质相似,所以分离的MSA淋洗液浓度不宜过高;MA^+^、DMA^+^、EA^+^的保留能力相近,高浓度淋洗液难以分离这3种离子,综合考虑分离度,选择在0~22 min设置淋洗液浓度为2 mmol/L; K^+^和TMA^+^的保留性质相近,综合考虑设置22.1~55 min的淋洗液浓度为3.5 mmol/L,保证两者的完全分离;Mg^2+^、Ca^2+^离子的保留性强,但也能完全洗脱。将淋洗液流速定为1.1 mL/min。优化后的色谱柱和梯度洗脱程序见1.2节,该条件下9种离子的标准物质色谱图见图3a。

### 2.2 方法学考察

#### 2.2.1 精密度、线性关系与检出限

将4种有机胺的标准储备液和5种阳离子的标准溶液配制成不同浓度的混合标准工作液。按1.2节中的色谱条件进行测定。以9种离子的质量浓度和色谱峰面积分别作为横坐标*X*和纵坐标*Y*,绘制标准工作曲线;取MA^+^、DMA^+^、TMA^+^、EA^+^质量浓度为0.04 mg/L, Na^+^、K^+^、Mg^2+^、Ca^2+^质量浓度为2.5 mg/L, N $H_{4}^{+}$ 质量浓度为10 mg/L的混合标准工作液测定6次并计算峰面积的RSD,见表1。结果表明:9种离子在各自相应的质量浓度范围内线性关系良好,相关系数(*r*)均不小于0.997;分别以信噪比*S/N*=3和*S/N*=10计算检出限(LOD)和定量限(LOQ), 9种离子的LOD为0.02~1.90 μg/L, LOQ为0.07~6.32 μg/L;各离子的RSD为0.08%~2.25%,精密度良好。根据采样时间和采样流速计算,得到大气PM_2.5_中9种阳离子的LOD为0.003~0.26 ng/m^3^, LOQ为0.01~0.88 ng/m^3^;取3个同样地点和时间的PM_2.5_采样滤膜,每个对半剪开,共6份,进行平行处理,连续进样,测定结果的RSD小于2%,方法重复性良好。

**表1:** 9种目标离子的线性范围、线性回归方程、检出限、定量限和精密度

Analyte	Linear range/(mg/L)		Linear regression equation	r	LOD/(μg/L)	LOQ/(μg/L)	RSD/%(n=6)
Na^+^	0.50-	20	Y=0.2662X-0.0100	0.9999	0.22	0.74	0.08
N	1.0-	30	Y=0.1465X+0.4670	0.9970	0.10	0.33	1.99
K^+^	0.25-	10	Y=0.1647X-0.0094	0.9999	0.38	1.27	0.32
Mg^2+^	0.25-	10	Y=0.4658X-0.0369	0.9999	0.61	2.02	0.58
Ca^2+^	0.25-	20	Y=0.2341X+0.0128	0.9992	1.78	5.90	0.59
MA^+^	0.0050-	0.20	Y=3.4791X-0.0091	0.9999	0.02	0.07	1.45
DMA^+^	0.0050-	0.20	Y=2.1343X-0.0088	0.9990	0.05	0.17	1.86
TMA^+^	0.0010-	0.050	Y=1.1653X-0.0016	0.9990	0.10	0.34	2.25
EA^+^	0.0069-	0.047	Y=0.0456X-0.0001	0.9990	1.90	6.32	1.15

*Y*: peak area of analyte, μS·min; *X*: mass concentration of analyte, mg/L.

#### 2.2.2 加标回收率

在PM_2.5_样品中分别加入不同体积固定浓度的9种标准离子溶液,再加入10 mL超纯水,得到每种离子低、中、高3个水平的加标溶液,每个水平平行处理6次,计算加标回收率(见表2)。结果显示,9种离子的加标回收率为93.2%~104%, RSD为0.25%~2.79%,说明该方法的准确性高,满足测试要求。

**表2:** 9种目标阳离子在PM_2.5_水溶液中3个水平下的加标回收率及RSD(*n*=6)

Cation	Background/ (mg/L)	Added/ (mg/L)	Recovery/ %	RSD/ %
Na^+^	4.4	2.2	99.9	0.25
		4.4	99.8	0.54
		6.0	101	1.15
N	2.0	1.0	99.1	1.78
		2.0	102	0.43
		3.0	100	0.98
K^+^	0.27	0.13	96.8	1.52
		0.26	101	0.63
		0.35	99.8	0.65
Mg^2+^	0.32	0.16	101	1.04
		0.32	101	1.17
		0.50	100	0.45
Ca^2+^	3.6	1.8	100	1.87
		3.6	100	1.26
		6.0	99.8	0.92
MA^+^	0.063	0.025	93.2	2.79
		0.060	99.6	1.48
		0.090	98.7	1.00
DMA^+^	0.065	0.030	98.8	2.23
		0.060	101	1.82
		0.090	101	1.84
TMA^+^	0.0056	0.0030	101	2.56
		0.0060	103	1.86
		0.0090	104	1.68
EA^+^	0.0087	0.0025	100	2.53
		0.0065	103	1.80
		0.012	101	1.46

### 2.3 实际样品分析

对采集的189个PM_2.5_样品按1.4节步骤处理,采用1.2节的色谱方法进行检测,得到样品的典型色谱图见图4。

**图4:**
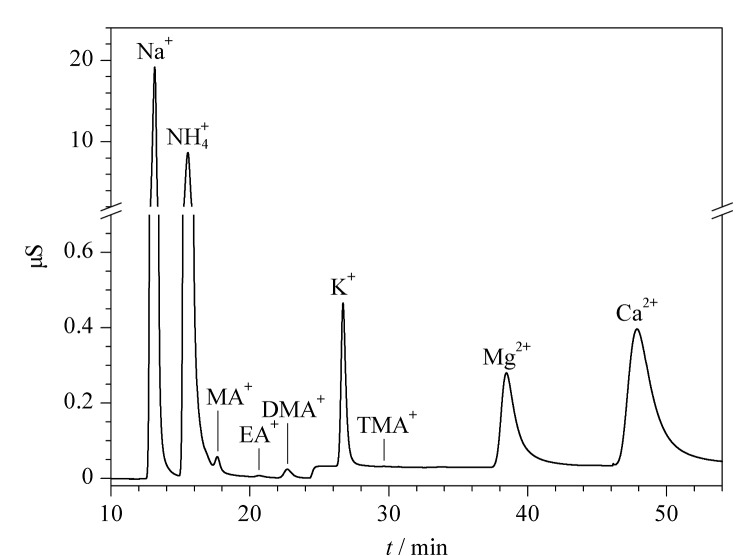
SH-CC-9分离大气颗粒物PM_2.5_样品溶液中9种阳离子的典型色谱图

根据公式(1)计算,得到大气PM_2.5_中阳离子和低级脂肪胺的检测结果,部分结果见表3。检测结果显示,采集的实际大气样品中都含有5种常见阳离子Na^+^、N $H_{4}^{+}$ 、K^+^、Mg^2+^、Ca^2+^,其中Na^+^、N $H_{4}^{+}$ 、Ca^2+^含量很高;含有少量部分或全部的4种低级脂肪胺甲胺、二甲胺、三甲胺和乙胺。在PM_2.5_样品的低级脂肪胺组成中,甲胺、乙胺、二甲胺含量较高,其中甲胺、乙胺的主要来源可能是机动车尾气排放^[30]^,表明采集区域内车流量较大,需要警惕尾气污染问题;同时PM_2.5_样品中三甲胺含量最少,而二甲胺浓度远低于3 mg/m^3^,使得促进颗粒物生成的几率大大降低^[31]^,从而减少了雾霾天气形成的可能。综合检测结果分析,采样区域低级脂肪胺的污染程度较低,对生活人群的危害较小。

**表3:** 部分大气颗粒物PM_2.5_样品中9种目标阳离子的含量

Sample No.	Na^+^	N	K^+^	Mg^2+^	Ca^2+^	MA^+^	DMA^+^	TMA^+^	EA^+^
1	3450.15	2423.74	288.00	141.90	887.35	0.49	0.67	0.25	3.42
2	3553.60	1977.85	368.33	179.47	1099.58	0.46	0.69	0.22	2.67
3	3791.32	2510.00	481.19	180.15	1133.92	0.53	0.76	0.25	<0.26
4	2282.81	1523.96	300.29	71.61	646.49	<0.03	0.65	0.21	1.04
5	3288.50	264.04	108.42	130.17	637.82	0.54	0.64	0.19	<0.26
6	3832.58	4475.63	542.54	129.46	844.83	0.53	0.60	<0.01	4.00
7	3861.39	2425.26	216.26	133.99	630.75	<0.03	<0.07	<0.01	1.43
8	3237.78	1771.75	261.07	110.22	639.65	0.54	0.78	0.22	<0.26
9	2677.17	1786.00	121.63	72.06	488.20	0.76	1.08	0.21	0.91
10	3116.08	4515.04	461.85	149.26	639.76	0.72	0.90	0.28	1.18
11	3215.10	2932.61	306.33	118.36	539.76	0.64	0.88	0.28	2.88
12	2265.15	2421.83	146.81	114.90	441.58	0.63	1.22	0.26	0.87

## 3 结论

本文建立了一种简单、灵敏、快捷的阳离子电导检测离子色谱方法,实现了同时对PM_2.5_中4种低级脂肪胺甲胺、二甲胺、三甲胺、乙胺和5种常规阳离子Na^+^、N $H_{4}^{+}$ 、K^+^、Mg^2+^、Ca^2+^的分析检测,尤其实现了高含量K^+^和痕量甲胺、乙胺和二甲胺的有效分离,从而可进一步检测环境大气中的阳离子组成和含量,实现对大气质量的监测。通过方法学考察验证了该方法具有很高的灵敏度、良好的重复性和准确度,对189个实际大气采样PM_2.5_样品检测结果表明,该方法能够同时满足大气PM_2.5_中痕量低级脂肪胺和高浓度阳离子的检测,对研究和监测大气中胺污染特征提供了简便可行的检测方案,其结果可为胺污染源的溯源提供依据,为雾霾的治理和人类健康提供有力的技术支持。
